# 

**Published:** 1896-01-25

**Authors:** 


CADBURY'S

COCOA

The standard of highest purity at present attainable."—*Lancet.*

### THE HOSPITAL

CADBURY'S

COCOA

NO ALKALIES USED.

LIBRARY
Index
S. G. O.

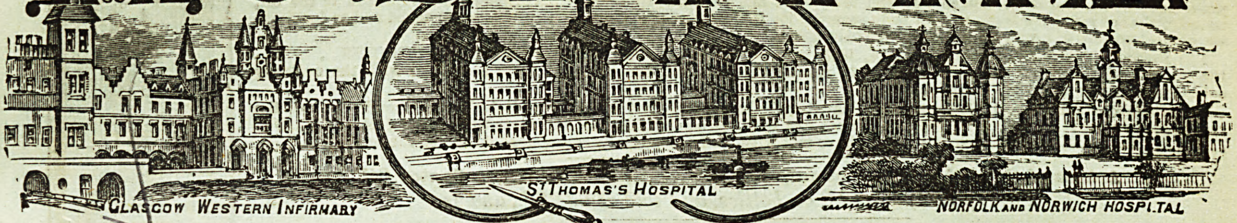

No. 487. Vol. XIX.
SATURDAY, JAN. 25TH, 1896.

**WITH EXTRA NURSING SUPPLEMENT.**

[Registered at the General Post Office as a Newspaper for transmission in the United Kingdom and Abroad.]

**PRICE TWOPENCE.**

Per Annum, 10s. 6d., post free.

Monthly Parts, 6d.; post free, 8d.

Ditto per Annum, 8s.6d., post free.

